# Psychological Impact of Acne Vulgaris on the Young Saudi Population

**DOI:** 10.7759/cureus.20509

**Published:** 2021-12-19

**Authors:** Amal Alqahtani, Wafa I Alsaab, Bader Altulahi

**Affiliations:** 1 Family Medicine, King Abdulaziz Medical City, Riyadh, SAU

**Keywords:** community mental health, scar, skin, quality of life, acne

## Abstract

Background

Acne is a chronic inflammatory skin disease caused by bacterial colonization that damages the pilosebaceous gland on the face and other parts of the body. It is one of the most frequent dermatologic diseases in the young population. Acne vulgaris is a devastating disease, and it has a significant impact on a patient's quality of life, influencing their self-esteem as well as their psychosocial development. This study aimed to explore the psychological symptoms associated with patients with acne, its impact on their quality of life according to their personal characteristics, and to raise the importance of observing and managing psychological symptoms during acne treatment.

Methods

In this cross-sectional study, data were collected through a paper questionnaire. The questionnaire was composed of two parts, the first includes data about demographics and general health while the second has details about the Dermatology Life Quality Index (DQLI); the Arabic validated version was used for data collection. Data were analyzed using SPSS version 20 (IBM Corp., Armonk, NY).

Results

According to the impact of acne on respondents' lives, 40.0% have no effect at all, 31.0% have small effects, 19.0% have moderate effects,9.0% have large effects, and 0.7% have extremely large effects.

Conclusions

Acne is a major problem that affects the quality of life of young patients. Its effect is significantly higher among less educated patients and those with a longer duration of disease.

## Introduction

Acne is a chronic inflammatory skin disease caused by bacterial colonization that damages the pilosebaceous on the face and other parts of the body. It is one of the most frequent dermatologic diseases in the young population [1].

Acne vulgaris has a significant impact on a patient's quality of life. It has a great impact on patients' self-esteem as well as their psychosocial development [2]. Patients and doctors are confronted with a plethora of over-the-counter and prescription acne treatments, making it difficult to choose the most effective treatment. Acne has a complex etiology that involves aberrant follicular keratinization, increased sebum production due to hyperandrogenism, Propionibacterium acnes growth, and inflammation [3].

Acne is thought to impact 9.4% of the global population, making it the eighth most common disease on the planet. Acne is most common in postpubescent teens, with males are being affected more than females especially with the severe presentation of the condition [4-5]. According to a Turkish study, acne is more widespread and severe in females, and it is linked to worry and despair [6]. Acne vulgaris was more common in females (200, 60%) than male students (133, 40%) in Egypt [7].

The majority of epidemiological studies on acne were conducted in the United States and the United Kingdom from the mid-to-late twentieth century, with the largest study being a population-based study involving more than 20,000 Americans. Nearly a third (312.4 per 1,000) of people or 60.6 million people in the United States, aged one to 74, have some form of skin pathology or one or more serious skin disorders [8]. According to Kilkenny et al., the overall prevalence rate of acne was 36.1% in Australia, with rates ranging from 27.7% in 10-12-year-olds to 93.3 percent in 16-18-year-olds [9]. Yahya H., who investigated 539 students aged 11 to 19, found that 90.7% of the students were suffering from acne [10].

Scarring and psychosocial distress are the two major acne consequences that last long after the active lesions have faded. Its start in adolescence may exacerbate the emotional and psychological difficulties that adolescence brings, as well as lead to developmental issues such as body image, socialization, and sexuality [11]. Acne, as a very noticeable skin problem, disrupts the lives of adolescents who are going through a variety of physical, intellectual, or emotional changes. While it is generally known that acne can cause melancholy and low self-esteem, the sociological evolution of adolescents in the twenty-first century is likely to exacerbate this effect [9,12].

A study conducted at the University of Miami Hospital found that post-inflammatory hyperpigmentation is more common on the face; however, it does not harm people's quality of life since they disguise it with makeup. Fifty-six point two percent (56.2%) of Qassim University students have acne, with no notable difference between boys and girls. The overall frequency of Acne vulgaris among teenage and young girls in Riyadh city (the capital of Saudi Arabia) was 68.2%. A study in Al-Khobar city showed that stress due to acne was more in females than males [13-14].

Therefore, it is interesting to ask whether the quality of life of patients with acne becomes negatively affected by the disease.

In this study, we aimed to explore the psychological symptoms associated with patients with acne and its impact on their quality of life according to their personal characteristics and to raise the importance of observing and managing psychological symptoms during acne treatment.

## Materials and methods

Procedure

In this cross-sectional study, data were collected through a paper questionnaire. The questionnaire was composed of two parts, the first includes data about demographics and general health while the second part has details about the disease and quality of life (DQLI). The validated Arabic version was used for data collection. Patients between 15 and 30 years of age who attended the dermatology department and primary health care at the King Abdul Aziz Medical City, Riyadh, for treatment of acne were the target population. Patients with other dermatological disorders, medical conditions, and mental diseases were excluded from the study.

The scoring of each question was as follows: 

· Very much 3 

· A lot 2 

· A little 1 

· Not at all 0 

· Not relevant 0 

· For Question #7, ‘prevented work or studying’ 3

The DLQI is calculated by summing the score of each question, resulting in a maximum of 30 and a minimum of 0. The higher the score, the more quality of life is impaired.

Interpretation of the DLQI scores: 

· 0-1 No effect at all on patient's life

· 2-5 Small effect on patient's life

· 6-10 Moderate effect on patient's life

· 11-20 Very large effect on patient's life

· 21-30 Extremely large effect on patient's life

The study variables were qualitative, with quantitative variables (e.g., age and acne duration) being categorized into groups. The main dependent variable in the present study was the quality of life of acne patients while the independent variables included patients’ personal characteristics (e.g., age, gender, educational status, marital status, number of children) and disease duration. Therefore, the main outcome point in the present study was the quality of life of acne patients.

The sample size was determined by maintaining an α error of 5% and a β error of 0.80 (study power of 80%) with a confidence interval of 95%, which was estimated to be 300 cases. These cases were randomly selected by convenience sampling. Ethical approval was obtained from King Abdullah International Medical Research Center in our institute. Written consent was obtained from the respondents regarding participation in this study.

After the collection of data, the Statistical Package for Social Sciences (SPSS version 20) was used for statistical analysis. A chi-square test was used to assess the association between patients’ demographics and the impact of acne on their quality of life. The level of significance was at p<0.05. The Helsinki ethical principles were fully followed in this research.

Ethics

The study protocol was reviewed and approved by the Medical Research Ethics Committee at King Abdullah International Medical Research Center’s (KAIMRC), with reference number RC20/090/R.

## Results

**Figure 1:**
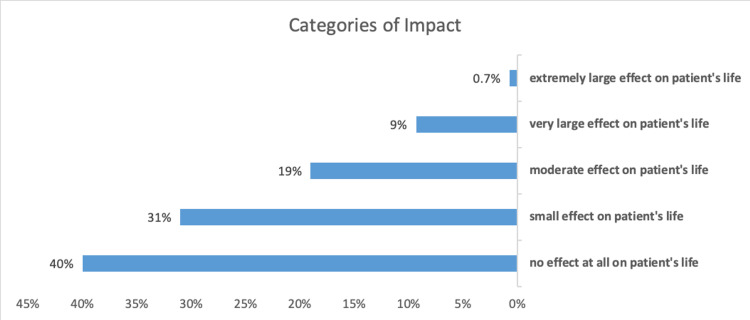
Categories of impact The figure shows the categories of the impact of acne on quality of life, with 40% of patients having no effect at all on their quality of life, 31% having small effects, 19% having moderate effects, 9% having large effects, and 0.7% having extremely large effects.

After analyzing the data of 300 respondents, 22.3% (67) were males while 77.7% (233) were females. Forty-four point three percent (44.3%) of respondents were between 20 and 25 years, 67.0% were educated at the university level, 84.7% were single, and 92.3% did not have any children. Fifty-six point three percent (56.3%) had two years or less duration while 43.7% had more than two years of duration. Ninety-nine point three percent (99.3%) presented with facial acne while 43% has acne manifestation on the back and 35% on the chest (Table 1). Regarding the consequences of the disease, 52.3% had pigmentation at the acne site, 38.70% had permanent scars while 12.7% had chronic diseases and 38% were scarred (Figure 1).

**Table 1:** Summarizes all participant information

Age		Frequency	Percent
	15 to less than 20	89	29.7
	20 to less than 25	133	44.3
	25 to 30	78	26.0
Literacy levels			
	Illiterate	2	0.7
	School	97	32.3
	university	201	67
Gender			
	male	67	22.3
	female	233	77.7
Marital status			
	single	254	84.7
	married	45	15
	divorced	1	0.3
You have children			
	yes	23	7.7
	no	277	92.3
Duration of recode			
	2 years or less	169	56.3
	more than 2 years	131	43.7
Location on face			
	yes	280	93.3
	no	20	6.7
location on chest			
	yes	105	35
	no	195	65
location on back			
	yes	129	43
	no	171	57
Family history of acne			
	yes	146	48.7
	no	154	51.3
Pigmentation			
	yes	157	52.3
	no	143	47.7
Scar			
	yes	114	38
	no	186	62
Chronic disease		Frequency	Percent
	yes	38	12.7
	no	262	87.3
	Total	300	100
Medications		Frequency	Percent
	yes	54	18
	no	246	82
	Total	300	100

Table 2 shows that the impact of acne on patients’ quality of life was significantly higher among those who were less educated (p=0.005) and with a longer duration of disease (p<0.001).

**Table 2:** Comparisons between demographical variables and impact categories This depicts that education and the duration of recode have a significant relationship with the categories of impact.

Crosstab								
			Impact Categories					Total
			no effect at all on the patient's life	small effect on the patient's life	moderate effect on the patient's life	very large effect on the patient's life	extremely large effect on the patient's life	
Age	15 to less than 20		34	27	19	9	0	89
			38.2%	30.3%	21.3%	10.1%	0.0%	100.0%
	20 to less than 25		50	44	25	12	2	133
			37.6%	33.1%	18.8%	9.0%	1.5%	100.0%
	25 to 30		36	22	13	7	0	78
			46.2%	28.2%	16.7%	9.0%	0.0%	100.0%
p= 0.812								
			Impact Categories					Total
			no effect at all on the patient's life	small effect on the patient's life	moderate effect on the patient's life	very large effect on the patient's life	extremely large effect on the patient's life	
Gender	Male		31	18	15	3	0	67
			46.3%	26.9%	22.4%	4.5%	0.0%	100.0%
	female		89	75	42	25	2	233
			38.2%	32.2%	18.0%	10.7%	.9%	100.0%
p=0.331								
Education			Impact Categories					Total
			no effect at all on the patient's life	small effect on the patient's life	moderate effect on the patient's life	very large effect on the patient's life	extremely large effect on the patient's life	
	illiterate		0	0	0	2	0	2
			0.0%	0.0%	0.0%	100.0%	0.0%	100.0%
	school		38	28	20	11	0	97
			39.2%	28.9%	20.6%	11.3%	0.0%	100.0%
	university		82	65	37	15	2	201
			40.8%	32.3%	18.4%	7.5%	1.0%	100.0%
p=0.005								
Marital status			Impact Categories					Total
			no effect at all on the patient's life	small effect on the patient's life	moderate effect on the patient's life	very large effect on the patient's life	extremely large effect on the patient's life	
	single		104	76	48	24	2	254
			40.9%	29.9%	18.9%	9.4%	.8%	100.0%
	married		16	17	8	4	0	45
			35.6%	37.8%	17.8%	8.9%	0.0%	100.0%
p=0.839								
Children’s			Impact Categories					Total
			no effect at all on the patient's life	small effect on the patient's life	moderate effect on the patient's life	very large effect on the patient's life	extremely large effect on the patient's life	
	yes		5	9	6	3	0	23
			21.7%	39.1%	26.1%	13.0%	0.0%	100.0%
	no		115	84	51	25	2	277
			41.5%	30.3%	18.4%	9.0%	.7%	100.0%
p=0.434								
			Impact Categories					Total
			no effect at all on the patient's life	small effect on the patient's life	moderate effect on the patient's life	very large effect on the patient's life	extremely large effect on the patient's life	
Q6_Duration_Recode	2 years or less		90	37	25	15	2	169
			53.3%	21.9%	14.8%	8.9%	1.2%	100.0%
	more than 2 years		30	56	32	13	0	131
			22.9%	42.7%	24.4%	9.9%	0.0%	100.0%
p=0.0001								

## Discussion

In this study, our aim was to determine the social and psychological effects of acne. We have observed that only 0.7% and 9% sustained a very severe or severe impact of acne, respectively, 19% had a moderate impact, while 31% and 40% had small or no impacts, respectively. This finding is concurrent with several other studies [1-8]. Studies conducted in Saudi Arabia reported that 64% of acne cases were mild, 30.1% were moderate, and only 5.9% were severe [12-14]. This finding is also consistent with findings from an Indian study, which indicated that Grade I acne was the most common, affecting 60.2% of participants However, a Chinese study found that 68.4% of acne patients had moderate acne. According to a study conducted in Riyadh, more than half of the cases (57.5%) had first-degree acne. Mild acne was seen in 53% of patients in Egypt [9,12-16].

In terms of gender and age, there was a trend of female preponderance to develop acne. Furthermore, across all age categories, ladies were more likely to be susceptible to acne than males. Less educated patients and those with a longer duration of acne experienced a significantly higher impact of acne on their quality of life.

Similarly, in Oman, findings of the Sultan Qaboos University study stated that the education level of acne patients was significantly associated with impact categories [17]. The observed significant impact of acne on QOL could be attributed to a variety of factors, including illness severity, cultural variances, individual perceptions, study design, and the evaluation tool.

**Figure 2:**
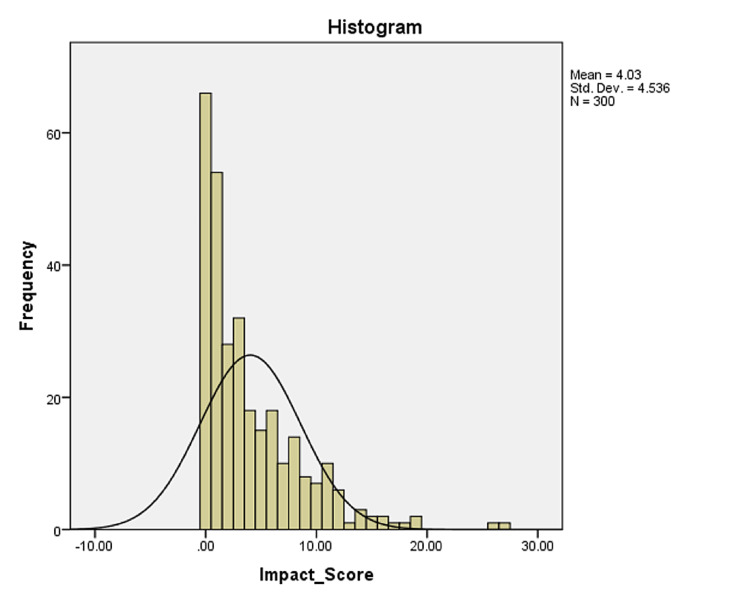
Histogram of impact score The figure shows that the mean impact score was 4.03, with the distribution being right-skewed. The median score was 3.

Acne and its consequences have a substantial impact on physical symptoms, emotions, social activities, study/work, and interpersonal relationships. According to our study, the average impact score of acne was around 4.0 (median = 3) (Figure 2), which is in alignment with other studies conducted in Saudi Arabia [3-5].

Lewis-Jones and Finlay stated that the mean scores for acne burden in the research were 4.3 and 5.7, respectively, confirming that patients with chronic skin illnesses, such as atopic eczema, psoriasis, and acne, had a lower quality of life than patients with other skin diseases. One study conducted in Brazil stated that the median score of quality-of-life questions was 4.02, which was similar to the findings of the study of Finlay et al. and higher than that of Walker and Lewis-Jones (score of 1.7). Despite the fact that this score indicates that the students were mentally affected in general, it still represents a moderate burden, owing to the significant frequency of mild acne among the adolescents investigated [18-20].

Study limitations

The study has some limitations such as not assessing risk factors such as food history, menstruation history, or family history. Moreover, the study followed a cross-sectional design, which is good for hypothesis generation, rather than hypothesis testing [21].

## Conclusions

Acne is a major problem that affects the quality of life of young patients. Its effect is significantly higher among less educated patients and those with a longer duration of disease. Therefore, effective preventive medicine programs are needed to help individuals deal with acne properly. This can be achieved by conducting different seminars and workshops to improve the quality of life. Encouraging people with acne to seek a professional consultation is important for improving their quality of life.
